# A phase II study of palbociclib plus letrozole plus trastuzumab as neoadjuvant treatment for clinical stages II and III ER+ HER2+ breast cancer (PALTAN)

**DOI:** 10.1038/s41523-022-00504-z

**Published:** 2023-01-06

**Authors:** Foluso O. Ademuyiwa, Donald W. Northfelt, Tracey O’Connor, Ellis Levine, Jingqin Luo, Yu Tao, Jeremy Hoog, Marie L. Laury, Tracy Summa, Trish Hammerschmidt, Zhanfang Guo, Ashley Frith, Katherine Weilbaecher, Mateusz Opyrchal, Rebecca Aft, Katherine Clifton, Rama Suresh, Nusayba Bagegni, Ian S. Hagemann, Michael D. Iglesia, Cynthia X. Ma

**Affiliations:** 1 Division of Oncology, Department of Internal Medicine, Washington University School of Medicine, St. Louis, MO 63110 USA; 2 Department of Medical Oncology, Mayo Clinic, Phoenix, AZ 85054 USA; 3 Department of Medicine, Roswell Park Cancer Institute, Buffalo, NY 14263 USA; 4 Siteman Cancer Center Biostatistics Shared Resource, Washington University School of Medicine, St Louis, MO 63110 USA; 5 Division of Public Health Sciences, Department of Surgery, Washington University School of Medicine, St. Louis, MO 63110 USA; 6 Genome Technology Access Center at the McDonnell Genome Institute at Washington University School of Medicine, St. Louis, MO 63110 USA; 7 Department of Surgery, Washington University School of Medicine, St. Louis, MO 63110 USA; 8 Department of Pathology and Immunology, Washington University School of Medicine, St. Louis, MO 63110 USA

**Keywords:** Breast cancer, Medical research

## Abstract

Patients with ER+/HER2+ breast cancer (BC) are less likely to achieve pathological complete response (pCR) after chemotherapy with dual HER2 blockade than ER−/HER2+ BC. Endocrine therapy plus trastuzumab is effective in advanced ER+/HER2+ BC. Inhibition of CDK4/6 and HER2 results in synergistic cell proliferation reduction. We combined palbociclib, letrozole, and trastuzumab (PLT) as a chemotherapy-sparing regimen. We evaluated neoadjuvant PLT in early ER+/HER2+ BC. Primary endpoint was pCR after 16 weeks. Research biopsies were performed for whole exome and RNA sequencing, PAM50 subtyping, and Ki67 assessment for complete cell cycle arrest (CCCA: Ki67 ≤ 2.7%). After 26 patients, accrual stopped due to futility. pCR (residual cancer burden—RCB 0) was 7.7%, RCB 0/I was 38.5%. Grade (G) 3/4 treatment-emergent adverse events occurred in 19. Among these, G3/4 neutropenia was 50%, hypertension 26.9%, and leucopenia 7.7%. Analysis indicated CCCA in 85% at C1 day 15 (C1D15), compared to 27% at surgery after palbociclib was discontinued. Baseline PAM50 subtyping identified 31.2% HER2-E, 43.8% Luminal B, and 25% Luminal A. 161 genes were differentially expressed comparing C1D15 to baseline. *MKI67*, *TK1*, *CCNB1*, *AURKB*, and *PLK1* were among the genes downregulated, consistent with CCCA at C1D15. Molecular Signatures Database gene-sets analyses demonstrated downregulated processes involved in proliferation, ER and mTORC1 signaling, and DNA damage repair at C1D15, consistent with the study drug’s mechanisms of action. Neoadjuvant PLT showed a pCR of 7.7% and an RCB 0/I rate of 38.5%. RNA sequencing and Ki67 data indicated potent anti-proliferative effects of study treatments. ClinicalTrials.gov- NCT02907918.

## Introduction

Human epidermal growth factor receptor 2 HER2 positive (HER2+) breast cancer represents 20–25% of invasive breast cancers. Several clinical trials, including NSABP B31, N9831, and BCIRG 006, established the role of adjuvant trastuzumab plus chemotherapy in patients with early HER2+ breast cancer^1–3^. Based on the APHINITY, NeoSphere, and TRYPHAENA studies, pertuzumab also became the standard therapy for patients with early HER2+ breast cancer in both the neoadjuvant and adjuvant settings^4–6^. As such, most patients with high-risk or node-positive HER2+ breast cancer are treated with neoadjuvant chemotherapy plus dual HER2 blockade with trastuzumab and pertuzumab regardless of estrogen receptor (ER) status.

Approximately 50% of HER2+ breast cancer is ER+, a population that comprises ~10% of all patients with breast cancer^7^. Unlike ER−/HER2+ tumors, which tend to be HER2-E by intrinsic subtyping, ER+/HER2+ breast cancers have a high proportion in the luminal A and luminal B categories, suggesting a different tumor biology^8–10^. Previous neoadjuvant trials show that patients with ER+/HER2+ tumors are less likely to achieve a pathological complete response (pCR) following neoadjuvant chemotherapy plus HER2-targeted agents. In addition, there is minimal effect of dual HER2 blockade therapy on pCR, unlike in those with ER−/HER2+ tumors^4,5,9^. For instance, in NeoSphere, the addition of pertuzumab to trastuzumab plus chemotherapy resulted in only a slightly higher pCR rate of 26% versus 20% in those with hormone receptor-positive disease. In those with ER− disease, pertuzumab added to trastuzumab and chemotherapy results in a much larger increased pCR rate of 63.2% versus 36.8%^4^. Furthermore, pCR rates differ significantly by intrinsic subtype in patients with HER2+ breast cancer. In CALGB 40601, those with HER2-E subtype achieve pCR rates of 70% while those with luminal A and luminal B achieve pCR rates of only 34% and 36%, respectively^9^. These findings suggest that those with ER+/HER2+ breast cancer are a group of patients in whom it is important to optimize novel therapies to improve outcomes and minimize toxicities.

Endocrine therapy plus trastuzumab is effective in advanced ER+/HER2+ breast cancer but results in a modest pCR rate. In both the TAnDEM and eLEcTRA trials, the addition of trastuzumab to aromatase inhibitors (AIs) improved time to progression (TTP) in patients with ER+/HER2+ metastatic breast cancer than in those who received AIs alone^7,11^. The phase II WSG-ADAPT trial assessed the efficacy of 12 weeks of neoadjuvant trastuzumab emtansine or trastuzumab with endocrine therapy in ER+/HER2+ early breast cancer and demonstrated a pCR rate of only 15.1% following the chemotherapy-free approach of trastuzumab with endocrine therapy^12^.

Cyclin-dependent kinase 4 and 6 (CDK4/6) inhibitors with endocrine therapy improve outcomes in advanced ER+/HER2− breast cancer^13–19^. Combined inhibition of CDK4/6 and HER2 results in synergistic reduction in cell proliferation, thus restoring sensitivity to HER2 inhibition in previously resistant cells^20^. The SOLTI-1303 PATRICIA trial showed that palbociclib plus trastuzumab plus endocrine therapy is safe and effective in patients with ER+/HER2+ advanced breast cancer^10^. Therefore, the purpose of PALTAN was to evaluate the pCR rate following neoadjuvant treatment with palbociclib plus letrozole plus trastuzumab in patients with ER+/HER2+ early breast cancer. Pertuzumab was not added to protocol therapy based on prior data from NeoSphere suggesting limited incremental benefit of dual HER2 blockade on pCR rates in patients with ER+ and/or PR+/HER2+ breast cancer.

## Results

### Patient characteristics

Between July 19, 2017, and March 31, 2020, 26 women with newly diagnosed ER+/HER2+ breast cancer were registered in the study. Median follow-up is 19.2 months. The patients’ characteristics are shown in Table 1. Median age is 59 years (range 32–78). Twenty-four (92.3%) were Caucasian and two (7.7%) were African American. Twenty-two (84.6%) and 4 (15.4%) patients had clinical stages II and III disease, respectively. Thirteen (50%) had clinically involved lymph nodes prior to treatment.

**Table 1:** Characteristics of the study population.

Characteristic	Total study population *N* = 26 (%)
Age at diagnosis, median (range)	59 (32–78)
*Race*	
Caucasian	24 (92.3)
Black	2 (7.7)
*Clinical stage*	
II	22 (84.6)
III	4 (15.4)
*Tumor grade*	
I	1 (3.9)
II	9 (34.6)
III	16 (61.5)
*Clinical node status*	
Positive	13 (50.0)
Negative	13 (50.0)
*Baseline PAM50 subtype* (*n* = 16)	
HER2 enriched	5 (31.2)
Luminal B	7 (43.8)
Luminal A	4 (25.0)

Baseline characteristics of the study population.

### Efficacy

All 26 patients were evaluable for efficacy. pCR (RCB 0) was achieved in 2 of 26 patients (7.7%; 95% CI 0.9–25.1%), and RCB 0/I was achieved in 10 patients (38.5%; 95% CI 20.2–59.4%). RCB I was observed in 8 patients (30.8%; 95% CI 14.3–51.8%), RCB II in 11 patients (42.3%; 95% CI 23.3–63.1%), and RCB III in 5 patients (19.2%; 95% CI 6.6–39.3%). The most common adjuvant therapy prescribed according to the discretion of the treating physician was trastuzumab, pertuzumab, carboplatin, taxane chemotherapy, and AI (Supplementary Table 1).

### Safety and tolerability

All 26 patients were evaluable for toxicity. 337 unique treatment-emergent adverse events (TEAEs) were reported in all patients (71.5% grade [G] 1, 19.3% G2, 8.6% G3, 0.6% G4); the most common were leukopenia (7.7%), neutropenia (7.1%), anemia (5.9%), and fatigue (5.6%). Sixteen patients (61.5%) had G1 fatigue, while 2 patients (7.7%) had G2 fatigue. Six patients (21.4%) had G1 diarrhea, while only 1 patient (3.8%) had G2 diarrhea. Table 2 shows all the G3/4 TEAEs that occurred in 19 patients (73.1%). Among the 19 patients, the incidence of G3/4 neutropenia was 50.0%, hypertension 26.9%, and leucopenia 7.7%. TEAEs (hypertension, ventricular tachycardia, pulmonary edema) leading to discontinuation of the regimen were reported in one patient (3.8%). Two patients (7.7%) had at least one SAE. Dose interruptions, reductions, or discontinuations due to toxicity occurred in 13 patients (50%). Among the 13 patients, palbociclib was interrupted in all 13, but reduced in only one patient. Trastuzumab was interrupted in two and discontinued in one patient. Letrozole was interrupted in two and discontinued in one patient. One patient developed progression after protocol therapy, but prior to surgery. No treatment-related deaths occurred.

**Table 2:** Treatment-emergent adverse events of ≥grade 3 in study participants.

All patients, *N* = 26 *N* (%)	
Grade 3/4 TEAEs	19 (73.1)
Neutropenia	13 (50.0)
Hypertension	7 (26.9)
Leucopenia	2 (7.7)
Lymphopenia	1 (3.8)
Colitis	1 (3.8)
Hypokalemia	1 (3.8)
Pulmonary edema	1 (3.8)
Ventricular tachycardia	1 (3.8)
ALT increase	1 (3.8)
AST increase	1 (3.8)
Colonic obstruction	1 (3.8)
SAEs	2 (7.7)
Fracture	1 (3.8)
Colonic obstruction	1 (3.8)
Pulmonary edema	1 (3.8)
Hypertension	1 (3.8)

Grade 3 or 4 treatment-emergent adverse events and serious adverse events in study participants.

### Patient-reported outcomes

**Fig. 1:**
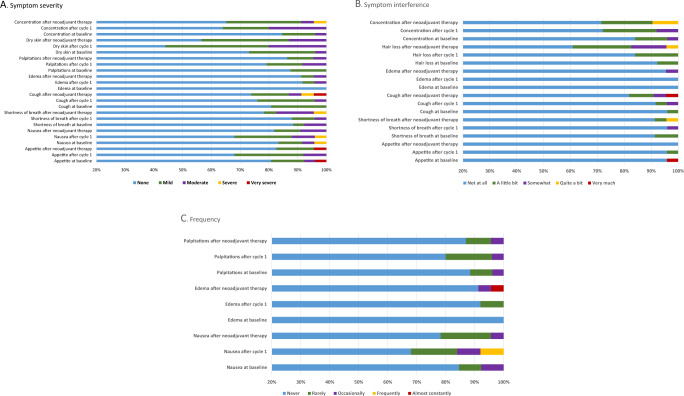
Patient-reported outcomes collected at baseline, after cycle 1, and after neoadjuvant therapy using NCI PRO-CTCAE. **A** Severity, **B** interference, and **C** frequency.

PROs were evaluated using an NCI PRO-CTCAE at baseline, after Cycle 1, and after completion of neoadjuvant therapy. Outcomes investigated included severity, degree of interference of symptoms, and frequency, including appetite, nausea, shortness of breath, cough, peripheral edema, palpitations, rashes, dry skin, hair loss, and concentration. The majority of participants had no or mild symptoms assessed from baseline to after neoadjuvant therapy (Fig. 1). There were no differences in appetite, nausea, shortness of breath, cough, edema, palpitations, rashes and dry skin, or concentration from baseline to end of C4. Patients had worsening hair loss from baseline to the end of C4 (*p* = 0.0315).

### Impact of palbociclib plus letrozole plus trastuzumab on cell proliferation

**Fig. 2:**
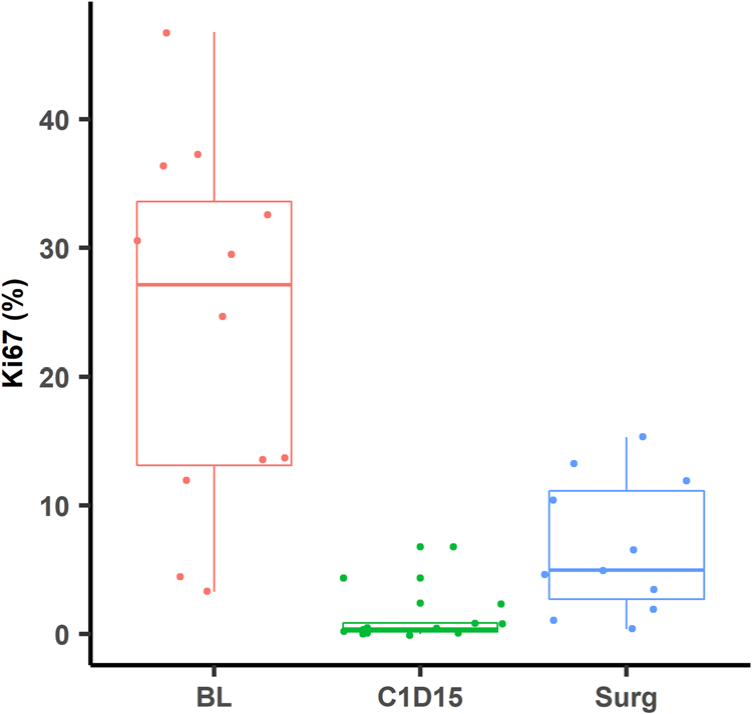
Boxplots comparing Ki67 scores in samples at baseline, C1D15, and surgery. The center line represents the median (middle quartile), the bounds of the boxes represent the upper and lower quartile, and the whiskers represent the minimum and maximum values.

**Fig. 3:**
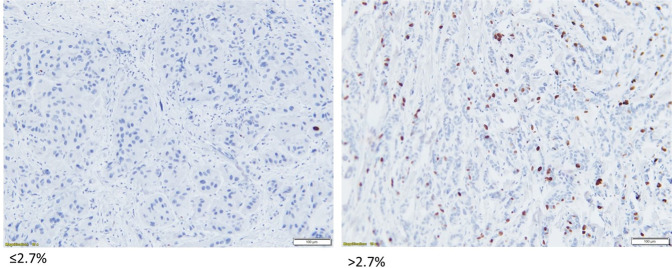
Image of tumor sample with Ki67 scoring of ≤2.7% and >2.7% by immunohistochemistry (IHC).

The effect of neoadjuvant treatments on cell proliferation was assessed by IHC analysis of percent Ki67 on FFPE tumor biopsies performed on 12 of 26 (46.1%) available samples taken at baseline, 18 (69.2%) samples taken at C1D15, and 11 (45.8%) samples available from the surgery. Analyses indicated complete cell cycle arrest (CCCA; Ki67 ≤ 2.7%) in 11 of 13 evaluable samples (84.6%) at C1D15 following palbociclib, compared to 3 of 11 samples (27%) at surgery after palbociclib had been discontinued, *p* = 0.0042 (Fig. 2). Median Ki67 was 27.1%, 0.38%, and 4.9% at baseline, C1D15, and surgery respectively. Figure 3 shows examples of tumor samples with Ki67 scoring of ≤2.7% and >2.7%.

### Molecular profiling

**Fig. 4:**
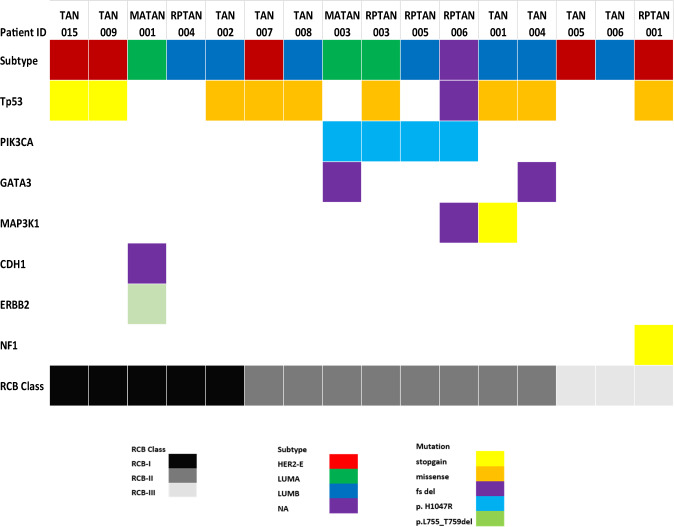
Tumor somatic mutations by WES occurring in at least three patient samples according to pathological status. The vertical axis represents the patient ID, tumor molecular subtype, and genes with somatic mutations. Each column represents a unique patient’s sample.

WES was performed on baseline samples from 16 patients with sufficient tumor DNA. The distribution of pathological responses in these 16 patients was similar to the entire cohort. RCB I, II, and III were observed in 31.2%, 50%, and 18.8% of patients, respectively. Somatic mutations occurring in at least three or more patient samples were observed in 18 genes. Figure 4 is a heat map of somatic mutations according to the pathological response. Genes with the most frequent mutations were *TP53* gene mutations occurring in 62.5% of the samples, and *PIK3CA* in 25%. Notably, all patients with *PIK3CA* mutations had more extensive residual disease with either RCB II or III.

**Fig. 5:**
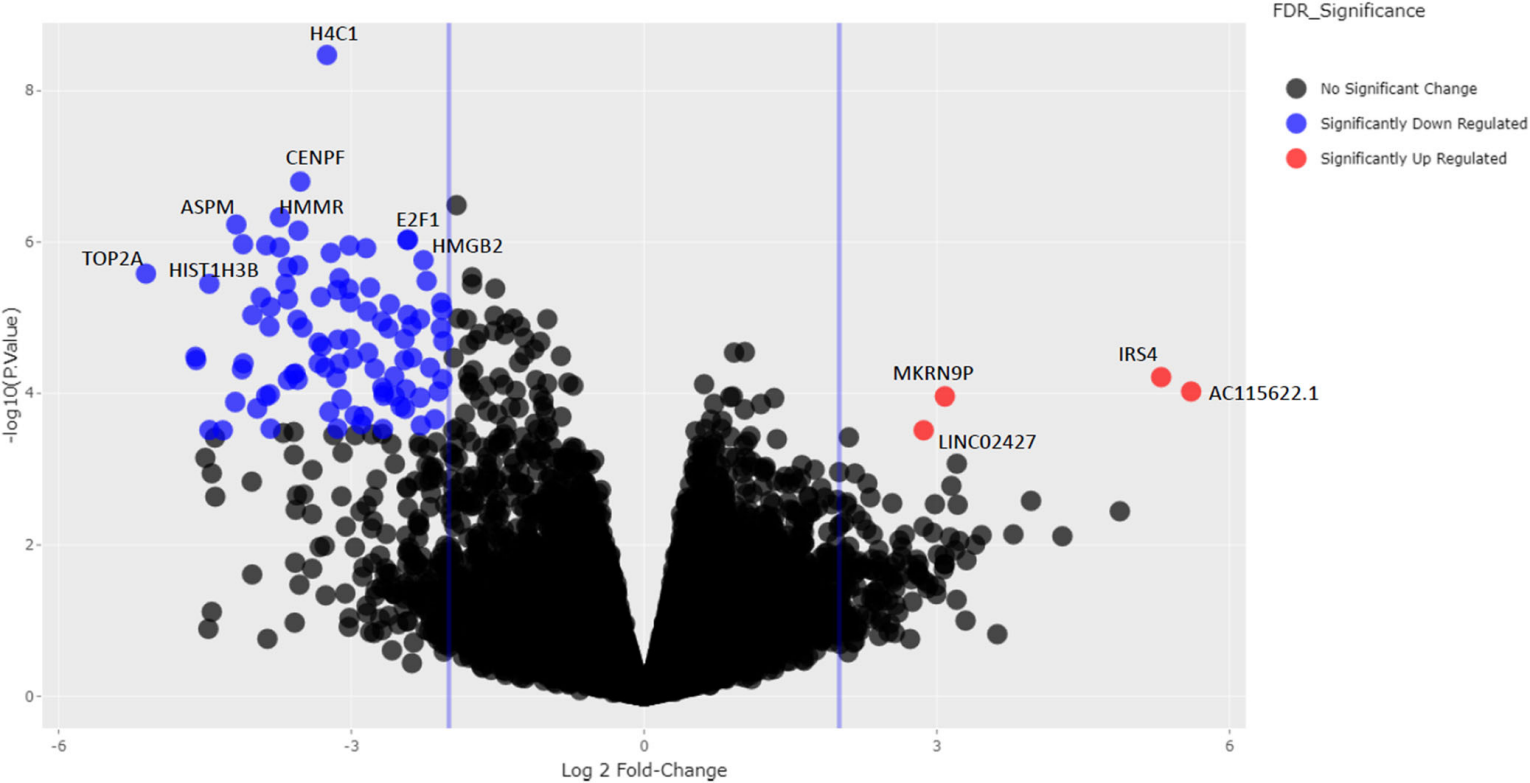
Differentially expressed genes at C1D15 versus Baseline. The volcano plot color codes the genes that are statistically significant and have a log2 fold change of +2 or −2.

**Fig. 6:**
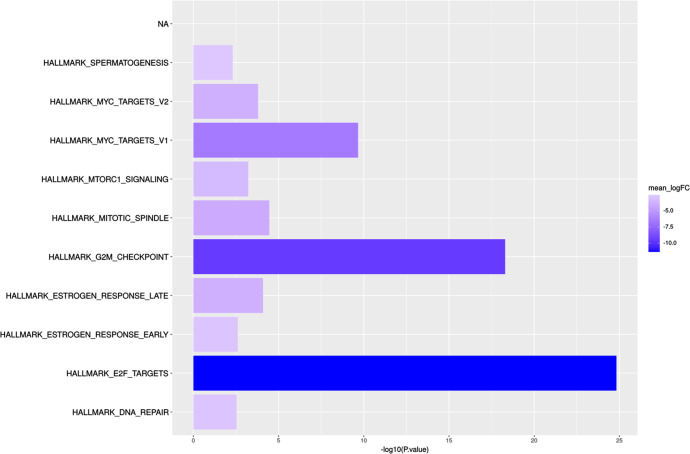
Significantly up- and down-regulated biological processes at C1D15 versus Baseline. Generally applicable Gene-set Enrichment analysis of Molecular Signatures Database Hallmark gene-sets comparing C1D15 and baseline samples.

RNA sequencing was performed on RNA isolated from 23 available tumor rich (>50% tumor cellularity) frozen biopsy samples collected at baseline (*n* = 16), C1D15 (*n* = 5), and surgery (*n* = 2) time points from 16 patients. Among the 16 baseline samples, PAM50 subtyping identified 5 HER2-E (31.2%), 7 luminal B (43.8%), and 4 luminal A (25%). All 5 samples assessed at the C1D15 time point were luminal A subtype (3 of these had switched from a baseline HER2-E subtype, and 2 had switched from a baseline luminal B subtype). The 2 samples assessed at the surgery time point were also luminal A subtype (1 had switched from a baseline luminal B subtype, and 1 remained luminal A subtype). The sample size was too small to assess for relationships between the PAM50 subtype and RCB categories. 161 genes were differentially expressed (FDR *p* < 0.05); 145 were downregulated and 16 upregulated comparing C1D15 to baseline. *MKI67, TK1, CCNB1, AURKB, and PLK1* were among the downregulated genes, consistent with CCCA for the majority of the samples at C1D15 by Ki67 (Fig. 5). Supplementary Table 2 is a list of all 161 up- and downregulated genes. Analysis of the Molecular Signatures Database Hallmark gene sets comparing C1D15 and baseline samples demonstrated downregulated biological processes involved in proliferation (E2F targets, G2M checkpoint, MYC targets, mitotic spindle), signaling (estrogen response, mTORC1 signaling), and DNA damage (DNA repair) at C1D15, consistent with the mechanisms of action of the study drugs (Fig. 6). E2F targets were higher in baseline samples of RCB II/III, compared to RCB I (FDR *p* = 0.042).

## Discussion

Several de-escalation approaches are being evaluated in patients with HER2+ breast cancer in order to avoid over-treatment while still maintaining treatment efficacy, and also to reduce the side effect burden of systemic therapy. In PALTAN, we showed that treatment with palbociclib, letrozole, and trastuzumab was feasible and well tolerated with no worsening in PROs. However, the trial was terminated early due to futility because of the observed pCR rate of only 7.7%. The low pCR rate of endocrine therapy plus HER2 blockade alone in ER+/HER2+ early breast cancer observed in PALTAN is also consistent with results from other trials. The PHERGain study evaluating de-escalating neoadjuvant therapy in patients with HER2+ breast cancer demonstrated a pCR rate of 32.3% in patients with ER+/HER2+ early breast cancer treated with neoadjuvant endocrine therapy, but with dual HER2 blockade utilizing trastuzumab, and pertuzumab^21^. This study also showed a pCR rate of 47.7% in the cohort of patients with ER+/HER2+ treated with standard docetaxel, carboplatin, trastuzumab, and pertuzumab. A similar pCR rate of 27% was observed in NA-PHER2, a neoadjuvant phase 2 study also utilizing a chemotherapy-free approach with six cycles of dual HER2 blockade with trastuzumab and pertuzumab plus five cycles of palbociclib and fulvestrant in ER+/HER2+ breast cancer^22^. It is unlikely that the difference in the number of neoadjuvant therapy cycles explains the difference in pCR rates between PALTAN and NA-PHER2. Likewise, in the TBCRC 006 trial, the pCR rate with dual HER2 blockade utilizing trastuzumab plus lapatinib and endocrine therapy was 21% in patients with ER+/HER2+ cancers^23^. In NeoSphere, the pCR rate was higher with trastuzumab and pertuzumab without endocrine therapy in patients with ER−/HER2+ cancers (27.3%) versus those with ER+/HER2+ cancers (5.9%)^4^. Taken together with other prior studies, these data confirm that patients with HER2+ breast cancer have a differential response to anti-HER2-based systemic therapy depending on ER status. Although pCR rates are lower for patients with ER+/HER2+ cancers following HER2 blockade with chemotherapy, the low pCR rates following chemotherapy-free approaches observed in PALTAN and several other trials suggest that chemotherapy remains a vital component for this group of patients. Other ongoing de-escalation trials, such as the CompassHER2-pCR (NCT04266249) and Decrescendo (NCT04675827) trials may maintain treatment efficacy while reducing the burden of side effects. These trials are both assessing reducing chemotherapy intensity while still maintaining dual HER2 blockade in patients with early HER2+ breast cancer.

There is a 4% rate of hypertension^24^ reported with trastuzumab; therefore, the 26.9% rate of G3 or G4 hypertension observed in seven patients on PALTAN was unexpected. One patient had undiagnosed hypertension leading to other complications including ventricular tachycardia and protocol treatment discontinuation. Rates of hypertension were not reported in the Phase II SOLTI-1303 PATRICIA Trial^10^. It is likely that the unexpected rate of hypertension seen in PALTAN is due to chance alone and not due to a synergistic effect of trastuzumab and palbociclib.

There is an ongoing debate about whether pCR is a valid surrogate of long-term outcomes in patients with ER+/HER2+ early breast cancer. A meta-analysis including seven randomized German trials showed that pCR in ER^_^/HER2+ breast cancer was associated with better long-term outcomes compared with no pCR, but there was no difference in ER+/HER2+ cancers^25^. However, other studies showed conflicting reports. Although the magnitude of the benefit was higher in ER− cancers, the Cortazar meta-analysis showed that pCR is a valid surrogate marker for long-term outcomes in patients with HER2+ breast cancer regardless of ER status^26^. Another recent meta-analysis with patients with HER2+ breast cancer confirmed that those who achieved a pCR also had better long-term outcomes versus those who did not, irrespective of ER status^27^.

Other early response evaluation criteria may help guide the selection of appropriate patients with ER+/HER2+ tumors for de-escalation approaches. Early reduction in the Ki67 index predicts responsiveness to endocrine therapy or endocrine therapy plus CDK 4/6 inhibition in ER+ breast cancer^28–30^. Neoadjuvant CDK 4/6 inhibitors with endocrine therapy has also been evaluated in early ER+/HER2- breast cancer and also result in molecular downstaging^30–33^. For example, NeoPalAna evaluated neoadjuvant palbociclib plus anastrozole for early ER+/HER2- breast cancer and showed that the addition of palbociclib enhanced anti-proliferation over and beyond endocrine therapy alone as evidenced by rates of CCCA^30^. The chemotherapy-free neoadjuvant regimen tested in NA-PHER2 with trastuzumab and pertuzumab plus palbociclib and fulvestrant was also effective at producing a reduction in Ki67 after only 2 weeks of treatment^22^. Consistent with other studies, PALTAN also demonstrated potent anti-proliferative effects of study treatments in the majority of tumor samples assessed for Ki67 and by RNA sequencing after 2 weeks of neoadjuvant treatment. Although small numbers, the observation that patients with *PIK3CA* mutations had more extensive residual disease generates the hypothesis that such patients with ER+ HER2+ disease may not be appropriate for endocrine-based de-escalation strategies. PHERGain assessed early metabolic responses using an FDG-PET response-adapted strategy^21^. In patients with ER+/HER2+ cancers treated with endocrine therapy with trastuzumab and pertuzumab, pCR rates were 35% in FDG-PET-responders versus 20% in FDG-PET-nonresponders. Therefore, combining early response evaluation with anti-proliferation indices and tumor imaging may guide patient selection in future trials for ER+/HER2+ breast cancer.

The small sample size and absence of a control group limit the interpretation of PALTAN. In addition, the correlative studies were performed on a subset of the study population further limiting the ability to learn from the translational correlative studies. For instance, the few samples that had RNA sequencing at surgery limited the analysis of pathways that were suppressed at C1D15 but rebounded at the time of surgery. Although the treatment regimen demonstrated potent anti-proliferative effects in the majority of tumor samples assessed for Ki67, very few achieved pCR. A treatment-induced shift towards a lower RCB class may have been more informative about the clinical relevance of the observed reductions in ki67. Unfortunately, the limited sample size precluded this analysis. The ongoing I-SPY2 neoadjuvant platform, which includes the evaluation of neoadjuvant endocrine therapy, may help shed light on this in the future. Most patients with the residual disease received postoperative adjuvant therapy at the discretion of the treating physician; this may affect long-term outcomes, but not the primary efficacy endpoint of pCR in PALTAN. Another limitation of this study was the omission of pertuzumab in protocol therapy. The rationale for omitting pertuzumab was based on prior from both NeoSphere^4^ and CALGB 40601^9^ suggesting the limited incremental benefit of dual HER2 blockade on pCR rates in patients with ER+ and/or PR+/HER2+ breast cancer. As the NA-PHER2 study with a higher pCR rate included dual HER2 blockade, evidence suggests that dual HER2 blockade remains a vital component of therapy.

In conclusion, this multicenter, open-label phase 2 study with palbociclib, letrozole, and trastuzumab did not meet its primary endpoint. Ki67 data and RNA sequencing indicated potent anti-proliferative effects of study treatments, despite significant heterogeneity of intrinsic subtypes. Until validated tools that provide useful information to identify patients who might not require chemotherapy are developed, most patients with high-risk or node-positive ER+/HER2+ early breast cancer require chemotherapy with trastuzumab and pertuzumab.

## Methods

### Patient eligibility

Eligible patients included pre- and post-menopausal women at least 18 years old, with newly diagnosed AJCC 7 clinical stage II or III ER+/HER2+ invasive breast cancer with complete surgical excision after neoadjuvant therapy as the treatment goal. Tumors were required to be at least 2 cm in one dimension by clinical or World Health Organization radiographic exam. Additional inclusion criteria included an Eastern Cooperative Oncology Group of ≤1, left ventricular ejection fraction ≥50%, corrected QT interval <480 milliseconds, and adequate organ and marrow function. Key exclusion criteria included prior treatment of the current breast cancer, indeterminate or HER2− status, inflammatory breast cancer, or a history of prior malignancy within the preceding 5 years.

### Study design and treatment

**Fig. 7:**
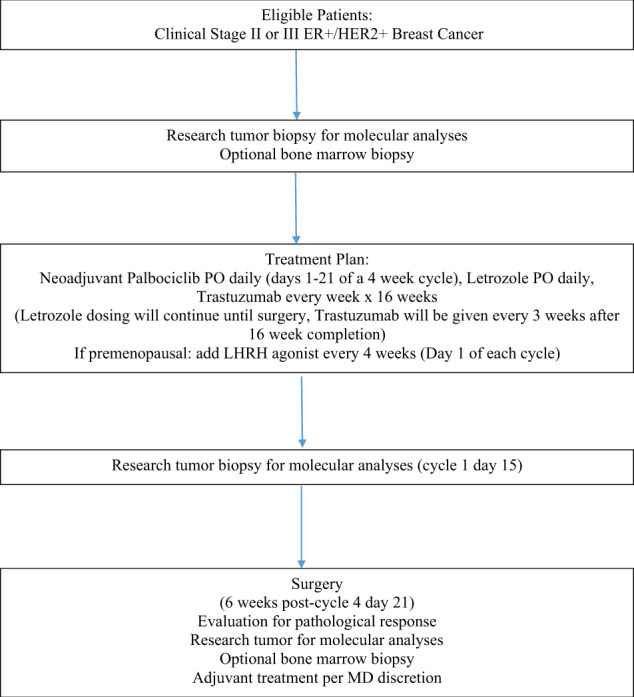
Clinical trial schema. Diagram of a clinical trial showing chemotherapy regimen and biospecimen collection time points.

The independent ethics committee at Washington University School of Medicine, St. Louis and each of the other participating sites (Mayo Clinic, Arizona, and Roswell Park Cancer Institute, Buffalo) approved this single-arm phase II study. The study was performed in accordance with the International Conference on Harmonization guidelines concerning Good Clinical Practice and the Declaration of Helsinki. ClinicalTrials.gov number is NCT02907918 and the registration date was September 14, 2016. All patients provided written informed consent. The study design is shown in Fig. 7. All eligible patients were prospectively assigned to single-arm treatment with letrozole (plus a GnRH analog if premenopausal), trastuzumab (or an FDA-approved biosimilar), and palbociclib for 4 cycles, each cycle consisting of 28 days (total of 16 weeks of neoadjuvant protocol therapy). Oral letrozole was administered at 2.5 mg daily on days 1–28, with the addition of a GnRH analog for premenopausal patients on day 1 of each cycle. Intravenous trastuzumab (or an FDA-approved biosimilar) was administered weekly on days 1, 8, 15, and 22 of each 28-day cycle with an initial loading dose of 4 mg/kg and subsequent doses of 2 mg/kg. Oral palbociclib was administered at 125 mg on days 1–21 of every 28-day cycle. Following 4 cycles of neoadjuvant therapy, palbociclib was discontinued to allow for blood count recovery prior to surgery; however, letrozole, GnRH analog, and trastuzumab continued until definitive surgery, which was within 6 weeks of completing 4 cycles of neoadjuvant therapy. Administration of further adjuvant anti-HER2 therapy, chemotherapy, and endocrine therapy was at the discretion of the treating physician. Patients who underwent breast conservation therapy received adjuvant radiation according to institutional practices.

Research tumor biopsies for correlative studies were obtained at baseline prior to neoadjuvant therapy, on cycle 1 day 15 (C1D15), and at the time of definitive surgery in patients with residual disease.

### Statistical consideration

The primary endpoint was the pCR rate in eligible patients treated with the palbociclib, letrozole, and trastuzumab combination. pCR was defined as no histological evidence of invasive tumor cells in the surgical breast sample and lymph nodes. Residual ductal carcinoma in the breast only was considered as pCR. Using a one-stage single-arm phase II design, we planned to enroll 48 patients in order to achieve 80% power at a 1-sided 0.05 significance level. Based on a prior trial evaluating 12 weeks of neoadjuvant trastuzumab emtansine or trastuzumab with endocrine therapy in ER+/HER2+ early BC, we assumed a null hypothesis of a pCR rate of 15% pCR^12^ and further hypothesized that a pCR rate of 30% would merit further investigation. Early stopping of the trial was based on unacceptable toxicity or futility. Once the first 20 evaluable patients were available, sequential futility monitoring for pCR was initiated. The trial was to stop for futility if we observed ≤2 subjects with pCR out of the first 20–26 evaluable patients.

Secondary objectives included safety, tolerability, and patient-reported outcomes (PROs). Fisher’s exact test was used to compare baseline and post-treatment changes in PROs. Exploratory objectives included assessment of tumor cell proliferation by Ki67 post 2 weeks of treatment, and assessment of intrinsic breast cancer subtype and tumor mutational profiles to correlate with treatment response.

### Residual cancer burden (RCB) scoring

Histologic slides from surgical cases post neoadjuvant therapy were reviewed to determine tumor bed size, percent neoplastic cellularity within the tumor bed, percent of residual tumor that was in situ, number of positive nodes, and largest lymph node deposit. RCB score and category were assigned by the MD Anderson method using a publicly available web calculator (http://www3.mdanderson.org/app/medcalc/index.cfm?pagename=jsconvert3).

### Ki67 immunohistochemistry and scoring

Five-micron tumor tissue sections were deparaffinized and rehydrated in a series of graded alcohols. Sections were treated with 3% hydrogen peroxide in methanol for 15 min to exhaust endogenous peroxidase activity, followed by antigen retrieval (Cat. S1699 Dako) for 20 min in a steamer. Sections were washed three times in phosphate-buffered saline (PBS) and blocked in protein block (Cat. X090930-2 Dako) for 10 min. The antibody of anti-Ki-67 Rabbit mAb (Cat. RM9106S0 Fisher Scientific) was diluted in 1:200 overnight at 40 °C. The slides were washed three times in PBS and incubated with EnVision™+ Single Reagents HRP, Rabbit (# K400311-1 Dako) for 60 min at room temperature. Substrate chromogen diluted in REAL substrate buffer (Cat. K5007 Dako) was then applied for 10 min with intermediate rinses in PBS. Subsequently, the slides were counterstained with Meyer’s hematoxylin for 30 s, dehydrated in ascending ethanol series, cleared with xylene, and coverslipped using a permanent mounting medium. Appropriate positive and negative controls were tested.

Ki67 index (percentage of tumor cells positive for Ki67 staining) was scored by visual point counting as described previously^34^. Briefly, photomicrographs of three randomly selected fields were taken at ×40 with a background grid and color printed (more fields to achieve the minimal cell count). Two observers count both the total number of tumor cells and the number of Ki67-positive cells that intersect with a grid line. Observer 1 starts the counting on grid line 1 and observer 2 starts on grid line 2. This process is repeated every third gridline for each observer. All the cells on the slide were counted if three fields could not be obtained. At least 200 total tumor cells were required. Combined Ki67-positive cells from both observers are divided by the combined total number of tumor cells to calculate the percentage positive Ki67 value.

### Whole exome sequencing (WES)

Scrolls (25–50 microns thick) of tissue were harvested from an OCT-embedded specimen with at least 50% tumor cellularity using a Leica CM1850 Cryostat and transferred to nuclease and protease-free 2.0 ml Eppendorf Safe-Lock microcentrifuge tubes on dry ice. Tissue scrolls were alternated among three tubes assigned for RNA, DNA, or protein extraction. Tissue scrolls designated for DNA extraction were washed by adding 1.0 mL of PBS and mixed by inverting the tubes 30 times. Samples were then centrifuged at 6000×*g* for 3 min and supernatants were removed. Qiagen’s QIAamp DNA Mini Kit (catalog # 51304) was used for DNA isolation according to the manufacturer’s protocol.

Genomic DNA samples were quantified using the Qubit Flourometer 3.0 or the VarioSkan Flash and ~25 ng of genomic DNA was used for sample assessment. When making the library, 100–250 ng of gDNA was fragmented using the Covaris LE220 to achieve a mean fragment size of ~200–250 bp. Libraries were then constructed using the KAPA Hyper Prep Kit (KAPA Biosystems, Cat # 7962363001) in conjunction with the automated Perkin Elmer SciCloneG3 NGS (96-well configuration). Libraries were then pooled at an equimolar ratio yielding up to 5 µg per library pool prior to the hybrid capture. Library pools were hybridized with the xGen Exome Research Panel v1.0 reagent (IDT Technologies) that spans 39 Mb target region (19,396 genes) of the human genome.

### RNA sequencing and analyses

Total RNA from fresh-frozen tumor biopsies at baseline and subsequent time points was extracted when at least 50% tumor cellularity was present^35^. Samples were prepared according to the library kit manufacturer’s protocol, indexed, pooled, and sequenced on an Illumina NovaSeq 6000. Briefly, total RNA integrity was determined using Agilent Bioanalyzer or 4200 Tapestation. Library preparation was performed with 500 ng to 1 μg of total RNA. Ribosomal RNA was removed by an RNase-H method using RiboErase kits (Kapa Biosystems). mRNA was then fragmented in reverse transcriptase buffer and heated to 94 °C for 8 min. mRNA was reverse transcribed to yield cDNA using SuperScript III RT enzyme (Life Technologies, per manufacturer’s instructions) and random hexamers. A second strand reaction was performed to yield ds-cDNA. cDNA was blunt-ended, had an A base added to the 3’ ends, and then had Illumina sequencing adapters ligated to the ends. Ligated fragments were then amplified for 12–15 cycles using primers incorporating unique dual index tags. Fragments were sequenced on an Illumina NovaSeq-6000 using paired-end reads extending 150 bases.

Illumina software and a custom python demultiplexing program were used for indexing reads. The resulting reads were aligned to the Ensembl release 76 primary assemblies with STAR version 2.5.1a^36^. Gene counts and isoform expression were derived by Subread:featureCount version 1.4.6-p5^37^. and Salmon version 0.8.2^38^, respectively. Genes were normalized and filtered within the R/Bioconductor package EdgeR^39^. Further RNA-Seq analysis was conducted utilizing the R/Bioconductor package Limma^40^. Differential expression analysis examined the differences between conditions. The most critical genes were identified via Limma voomWithQualityWeights and analyzed with weighted gene correlation network analysis and the R/Bioconductor package WGCNA^41^.

## Supplementary information

Supplementary Tables Legend

## Data Availability

The whole exome sequencing and RNA-seq data for this study have been deposited in dbGAP under accession number phs003147.v1.p1. The remaining data generated in this study are available upon request from the corresponding author.
